# Challenges facing clinical midwifery education in Iran

**DOI:** 10.1186/s12909-022-03485-6

**Published:** 2022-05-26

**Authors:** Maryam Hajiesmaello, Sepideh Hajian, Hedyeh Riazi, Hamid Alavi Majd, Roya Yavarian

**Affiliations:** 1 Department of Midwifery, Urmia Branch, Islamic Azad University, Urmia, Iran; 2 Department of Midwifery, School of Nursing and Midwifery, Shahid Beheshti University of Medical Sciences, Tehran, Iran; 3 Department of Biostatistics, School of Allied Medical Sciences, Shahid Beheshti University of Medical Sciences, Tehran, Iran; 4 Department of Psychiatry, Urmia University of Medical Sciences, Urmia, Iran

**Keywords:** Midwifery, Undergraduate medical education

## Abstract

**Background:**

Delivering high-quality midwifery services requires a professional, educated and competent workforce. The challenges of clinical training and education for midwives in Iran have prevented midwifery students from fully gaining the clinical competency required of midwifery graduates.

**Methods:**

This qualitative study was conducted to identify and explain the challenges of clinical training for midwives in Iran and to determine their underlying factors within the sociocultural and educational context of this country. Data were collected from a purposive sample in a western province of Iran, which included clinical midwives working in public and private maternity units, midwifery instructors working at educational institutes, and midwifery students. After receiving an ethics approval for the project and informed consent from the participants, data were collected through focus group interviews held with midwifery students (*n* = 9) and semi-structured interviews held with midwifery instructors (*n* = 6) and clinical midwives (*n* = 7). Data were then analyzed using the framework proposed by Graneheim and Lundman using MAXQDA-10.

**Findings:**

The analysis of the data led to two themes: “Discriminatory approach in the health system” and “Professional nature of midwifery”. The noted discrimination was caused by the insecure position of midwives in the health system, inequalities related to education and training opportunities, and the demotivation of midwives. The professional nature of midwifery discussed the community in transition, functional paradoxes and high-risk labor.

**Conclusion:**

The findings revealed numerous challenges facing clinical midwifery education and training in the study setting, which may in part be explained by the sociocultural context of maternity services in Iran. The learning opportunities provided to midwifery students should be improved by making significant revisions to the structure of clinical settings where students are placed. Tackling discrimination against a profession and its students is essential, and it is equally important to value the contributions of midwifery students and midwives to their practice and their efforts to ensure safe maternity care for women and newborns. The quality of the clinical learning environment must therefore be improved for this group, and the active participation of competent and autonomous midwifery instructors in this environment can have a facilitatory role.

## Background

With a key role in maternal and neonatal health, quality midwifery services can be provided by recruiting professional workforce [1]. According to the International Confederation of Midwives (ICM), midwives play key roles in providing specialized primary maternal care during pregnancy, labor, natural childbirth and postpartum period [2]. Evidence also suggests their key role in achieving the Millennium Development Goals, and their contribution to reducing maternal and neonatal mortality and morbidity is crucial as per the next decade’s Sustainable Development Goals (SDGs) [3]. Moreover, providing quality clinical education opportunities is crucial for educating students of medical sciences such as midwifery students.

Training midwives while aiming at their empowerment and clinical competence improvement thus lays the groundwork for providing safe, evidence-based and standard maternal care [4]. The WHO developed the Global Strategic Directions for Strengthening Nursing and Midwifery 2016–2020, and updated it in 2021–2025 (SDNM) and emphasized investment on nursing and midwifery workforce for ensuring acceptable, accessible, available, and high-quality health services. This planning should encompass recruitment of more service providers, improvement of quality and provision of resources and equipment at the workplace. High-quality care requires evidence-based education, regulations and practical standards [5].

The ICM mainly aims at the global promotion of midwifery through training midwives based on international standards. It also emphasizes the need for gaining basic clinical competence in providing evidence-based, standard high-quality midwifery services for women and families [5]. Despite the differences in the midwifery curriculum among countries, specific rules and standards are commonly applied to specialized theoretical and clinical education [6]. Socioeconomic, political and cultural factors, however, constitute obstacles to achieving the targets of sustainable high-quality midwifery education programs in different communities. Midwifery educators therefore constantly face these challenges in providing future professional midwives with education opportunities and essential maternal and neonatal care skills [3]. Research also suggests the failure of current clinical education programs to enable midwifery students to acquire clinical competence and skills [7]. A mixed methods study of intervention levels in different countries based on the SDNM developed by the WHO found only 19% of 35 studied countries to have thoroughly implemented this strategy in all domains. The programs of the first domain of this strategy (education) were completed in only 25% of the countries. This domain aimed at educating motivated and competent midwives and nurses at all levels of different services in responsive and efficient health systems [8].

In recent decades, maternity services in the labor wards of Iran have mostly been organized by the medical model, and midwives work under the supervision of obstetricians, as more than 50% of deliveries in Iran are performed by cesarean section [9]. Medicalizing health care for low-risk pregnancies has diminished midwives’ role in low-risk natural childbirth [10]. The policies adopted to promote natural childbirth after the 2014 implementation of the Health Transformation Plan in Iran included free-of-charge natural childbirth in state-run hospitals and holding childbirth preparation programs by midwives and physiological childbirth workshops for empowering midwives; nevertheless, midwives still face restrictions in providing midwifery services, especially those related to natural childbirth. Natural childbirth is performed by gynecology/obstetrics residents in teaching hospitals and midwives play a minor role in providing care during labor and birth. In non-teaching hospitals, midwives are not permitted to independently manage low-risk natural childbirth and supervised by specialists. In other words, obstetricians/gynecologists assume responsibility for the majority of childbirth cases in hospitals [10]. In the absence of referral systems and birth centers in cities and illegal home birth, childbirth must be absolutely performed in hospitals [11, 12]; nonetheless, low-risk birth is performed by midwives and midwife-oriented services are provided in birth centers in a few rural areas with no quick access to hospitals. The only urban birth center in Iran currently located in Urmia, West Azarbaijan Province provides midwifery-led services and refers pregnant women to a teaching hospital in case of complications.

Undergraduate midwifery education in Iran involves four years of theoretical, practical and clinical courses at schools of nursing and midwifery or schools of medicine. Admission to undergraduate midwifery programs is based on a competitive national exam. Clinical courses, including childbirth, are directly performed in hospital wards by midwifery instructors, who the majority of whom are faculty members. Their main task is midwifery students’ education at faculty and hospitals including labor wards during each semester. Faculty members assume sole responsibility for supervising the performance of the students and their acquisition of clinical experience. In some conditions such as faculty member shortage, a few experienced midwives of labor wards in teaching or public hospitals, may be recruited by the faculties to train and supervise the senior midwifery students as mentors. However, they are not assumed as official midwifery instructors, they involve the students’ educational process, encounter their clinical learning problems and feedback them to the head of midwifery department of the faculty if needed.

Despite teaching midwives in Iran for over a century, educating qualified midwives with university education in over 60 universities, the existing higher education opportunities [8] and alignment of midwifery education programs in this country with the recommendations of the ICM [10], certain obstacles to teaching clinical midwifery skills have caused major problems, especially in recent years.

A review study conducted in Iran categorized the educational needs of midwives as knowledge and practice, communication skills, occupational rules and regulations and religious rules [11]. A cross-sectional study revealed the unsupportive atmosphere of midwifery clinical education, emphasized the experience of disrespectful treatment of the learners and thoroughly recommended further investigations of the causes of these problems [12]. A qualitative study conducted in a school of nursing and midwifery in Iran reported inappropriate educational atmosphere, limited resources and inefficient midwifery curriculum as the main midwifery education issues [13]. Given the limitations of existing studies in this context, investigating the main challenging dimensions of midwifery education and their underlying factors and the causes of growing numbers of midwifery education problems still requires an in-depth exploration. The present qualitative research was therefore performed to explain the challenges facing clinical midwifery education from the perspectives of midwifery graduates working in maternity wards and midwifery instructors and students as the end users using a sample collected from two midwifery schools in Iran. The main dimensions of these challenges and their underlying factors were also identified to help lay plans for eliminating the obstacles, quantitatively and qualitatively improving clinical midwifery education and promoting the quality of midwifery care services.

## Methods

### Design and participants

The present qualitative study employed a conventional content analysis to explain the challenges facing clinical midwifery education in Urmia, the capital of West Azarbaijan, Iran from May 2020 to May 2021. The participants of this study were a number of midwifery students, midwives working in maternity wards and clinical midwifery instructors, mainly academic members, who met the following inclusion criteria: Having a bachelor’s or higher degree in midwifery, work experience in the labor and delivery department, being a junior and senior midwifery student in an undergraduate midwifery program in one of two medical schools, and volunteering and being able to share their experiences and transfer them to the researcher. The exclusion criterion was the participant's refusal to continue participating in the study at any time. Purposive sampling was used to select the participants while observing maximum variation in terms of demographic characteristics. Experienced midwives with ample knowledge were selected from the maternity wards of public teaching, public non-teaching and private hospitals and a maternity center providing midwife-centered services.

### Data collection

The participants selected through phone contact or presenting to two midwifery schools and three hospitals were briefed on the study objectives, provided primary consent and then agreed on the time and place of the interviews. The interviews were conducted in hospital or the school of nursing and midwifery mainly based on the participants’ preference. The data were collected through individual in-depth interviews with midwives and FGDs in groups of 3–6. The interviews lasted 45–75 min.

The interviews and FGDs were performed after obtaining written consent from the participants to record their voice, assuring them of the confidentiality of their information and emphasizing the exclusive access of the researchers to the audio files.

Semi-structured interviews conducted using an interview guide developed by the research team began with an open-ended question on the interviewee's view about "the main challenges of clinical midwifery education". Ambiguities were elucidated by asking the participant probing questions.

To warm up the FGD, the students were first asked about their personal details, the most common problems they had experienced in clinical education and potential causes of these problems. To avoid confusion after each interview and FGD, the audio files were transcribed by the researcher at the first opportunity and the interview text was analyzed before the following interview. The interviews continued until reaching data saturation. The data obtained from 13 individual interviews and two FGDs in two groups of three and six were ultimately analyzed. The first author (M.H.) conducted all the interviews.

### Data analysis

The data were analyzed using the conventional content analysis based on the steps proposed by Graneheim and Lundman [14]. Content analysis is an analytic approach that aims at explaining a phenomenon. Qualitative content analysis helps acquire knowledge and comprehend the study phenomenon and thus identify challenges. This approach is used to break down data into semantic units. In fact, the central feature of content analysis is to compress a large number of words in a text into smaller categorized content through systematic classification, coding and identification of themes [15]. The present research employed conventional or inductive content analysis given the limited information about the challenges perceived in clinical midwifery education and the lack of relevant theories and backgrounds. A new insight into these challenges was therefore obtained through engaging with the data and extracting their categories through inductive reasoning [16].The steps of the content analysis included:

- Reviewing the interview transcripts several times to acquire a general understanding.
- Reading the data verbatim to extract codes from the text.
- Preparing the list of codes.
- Classifying the codes as categories based on their similarities and differences.
- Clustering the categories and extracting the themes.
- Writing the definition of each category and subcategories.
- Preparing a report and presenting examples of each category.

Table 1 shows an example of the theme and descriptions used in the data analysis process. Coding and emerging of categories and themes were performed in MAXQDA 10.

**Table 1:** Examples of the theme and descriptions used in the analysis process

Theme	Category	Subcategory	Code	Quote (example)
Discriminatory approach in the health system	educational inequalities	Conflict between midwifery and residency	The priority of residency education	“They take us to hospitals that seem assigned to training doctors, and midwifery students can’t do anything there.”
			Unclear position of midwifery students	“It should be explained to them, because our position is not well defined in the health care system; what is our role? Well, if there is no need for midwives in this system, why are midwifery students are accepted?”

Also, The five criteria proposed by Guba and Lincoln used under the supervision of the research team to confirm the rigor and trustworthiness of the data included credibility, dependability, transferability, confirmability and authenticity [17].

## Findings

The mean age of the participating midwives was 39.30 ± 9.46 years and their mean work experience in maternity wards 14.53 ± 8.88 years. The mean age of the students was also 24 ± 1.22 years and their majority were a senior. Table 2 presents the demographic details of the participants.

**Table 2:** Details and distribution of participants

Participants		Number	Mean age	Marital status frequency (%)	Mean work experience/education (year)
Midwives	Midwives working in maternity wards	7	39.30 ± 9.46	Married: 9 (69.23) Single: 4 (30.76)	14.53 ± 8.88
	Midwifery instructor	6			
Midwifery students	Focus group 1	6	24 ± 1.22	Married: 2(22.2) Single: 7(77.7)	Year 3: 3 students Year 4: 6 students
	Focus group 2	3			

**Fig. 1:**
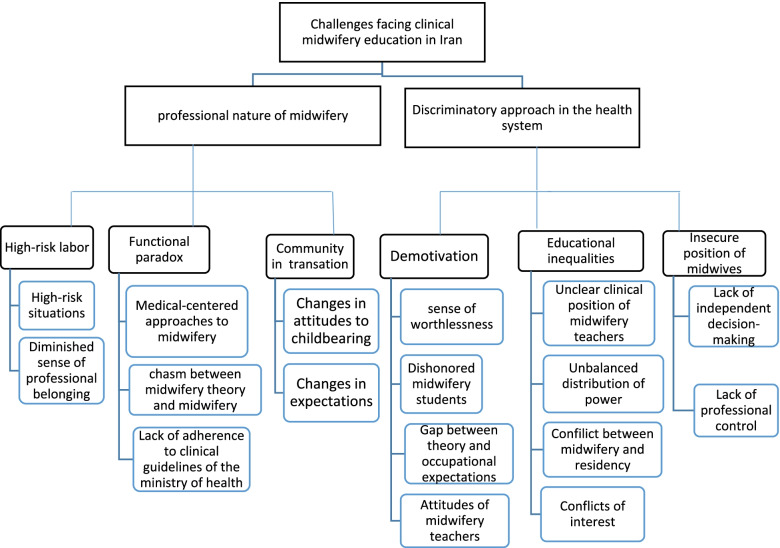
Conceptual model of the themes, categories and subcategories of challenges facing clinical midwifery education in Iran

Forty-nine final codes, excluding the repeated ones, extracted from the interview texts were placed in sixteen subcategories, six categories and two themes (Fig. 1). The main themes included “Discriminatory approach in the health system” and “ Professional nature of midwifery”.

### Discriminatory approach in the health system

According to the participants, discriminatory attitudes toward the midwifery profession in the health system caused the categories of challenges facing clinical midwifery education to be included in this theme. This theme comprised three categories, i.e. “Insecure position of midwives”, “educational inequalities” and “Demotivation”.

### Insecure position of midwives

The majority of the participants identified the unstable position of midwives in the health system. Despite the numerous applications of midwifery and its significant contribution to pregnancy care and conducting a low-risk childbirth, this profession has been largely neglected in the health system. The subcategories of this main category included “Lack of independent decision-making " and “Lack of professional control”.

Midwifery is a demanding profession. The responsibility of midwives require that they prevent interference in natural childbirth and promote decision-making by pregnant women and their participation in childbirth by providing appropriate information and recommendations. The atmosphere of labor rooms and dominance of obstetricians, however, cause their experiences to be neglected.

“Today, our profession has been made totally dependent on obstetricians, and we have been deprived of working independently. This has diminished our occupational position in the community. Job prospects of this profession also don’t look good.” (A midwifery instructor).

The majority of the participants were dissatisfied with their limited authority and lack of professional control and highlighted their insecure occupational position. Although midwifery has been organizationally subcategorized as nursing, midwives are neither regarded as nurses to act according to doctors’ instructions nor they are practically independent. In other words, midwives have been long deprived of their professional identity.

An academic member in midwifery said, “*Why hasn’t the position of midwife been defined? I have to train the student, who will return and help this system. Why hasn’t her position defined*?.”

### Educational inequalities

As a dimension of discriminatory attitudes toward the midwifery profession, “educational inequalities” was inferred from the comments of the participating midwifery students and instructors. "Unclear clinical position of midwifery instructors" was a subcategory of this category. According to the participants, the instructor’s clinical competence in maternity wards is ignored and they are required to comply with the ward’s routine procedures; meanwhile, they are not supported by their peers in a way that midwifery students and instructors are judged and blamed by their colleagues in cases of a traumatic childbirth in mothers under their care. Most obstetricians also refuse to accept responsibility in these cases or reluctantly perform specialized procedures.

“It is terrible that the delivery I perform should be in the presence of one of my previous year’s student as a novice in this hospital. The gynecologist sends this student and tells her to stand over me, while I am performing delivery with a student. This person was my student one or two years ago." (A midwifery instructor).

Complaining of the lack of clinical respect and limited welfare facilities for instructors, an experienced instructor said:

“Residents have made the room untidy; interns opened and used the gloves and didn’t put them in the trash bin; yet, it is the midwifery student and instructor who are interrogated, which is psychologically difficult to accept. I don’t know what their problem is with the instructor. It is truly annoying. We don’t have even a room or a closet in the hospital. There isn’t a chair for the instructor to sit in the labor room. I am there from 8 am to 1.30 pm; where am I supposed to sit?” (A midwifery instructor).

"They (staff) should cooperate more with us (students and instructors), because we are doing their job and reducing their workload, but everything we do there is ignored. Does it make any difference to be or not to be there? Our position has not been defined in health care system, even though we do the work of the whole ward and deliver as well.” (A midwifery instructor).

“Unbalanced distribution of power” constituted another subcategory. Some participants believed that midwives were in the minority in both the clinic and education settings. Poor legislation, especially as the non-participation of beneficiaries in decision-making and planning for midwifery, was another topic in this subcategory.

“We are in the minority and not properly supported. It is terrible to be in the minority; we are a subgroup of a subgroup of a medical discipline. We are in the minority even in the nursing school and the numbers of their students and faculty members (nursing department) are four and five times ours, respectively. All the managers, including head and vice chancellor of the faculty, are selected from nurses. We are therefore alone everywhere, in both the clinical setting and the faculty.” (A midwifery instructor).

Poor regulations on the distribution of power among organizational structural factors and clinical education mainly cause disadvantages to midwifery education, especially in labor rooms. An instructor said, “*Many of our problems are rooted in enacted regulations. Midwives are under so much pressure. There is a weak governance for midwifery. We are a group without the necessary position either at hospital management and higher management levels or at the ministry level.*” (A midwifery instructor).

According to the midwives, instructors and students, the conflict between midwifery and residency was a subcategory that was mainly formed by sharing the educational setting between midwifery students and gynecology residents. In this setting, gynecology residents are prioritized in terms of education. Although midwives play key roles in providing quality services at the first level of the health system, educational inequalities has deprived midwifery students of an effective education.

“Another problem is that they take us to hospitals that seem assigned to training doctors and midwifery students cannot do anything there. Perhaps, I would not be mistaken if I said the hospital is doctor-oriented.” (A student).

“Many times, I went to such a trouble to have a resident do the delivery. I conveyed this to the head of the department many times. Meetings were held several times. Letters were written. It will be put right over a period. Instructions were written and hung on the wall. Minutes were written that residents and midwifery students should do deliveries every other time. Yet, it was up to their taste. How much strength does one have to haggle over every delivery? It should be explained to them, because our position is not well defined in the health care system; what is our role? Well, if there is no need for midwives in this system, why are midwifery students are accepted?” (A midwifery instructor).

“Conflicts of interest” was another subcategory of “educational inequalities”. Midwives, physicians and gynecology residents sharing their financial benefits and nurses managing the midwifery profession significantly contribute to challenges in this subcategory. A midwifery instructor working in the hospital said,

“We are hurt by both gynecologists and the nursing team. Defining midwifery as the sole provision of care, they say we can also do it and midwifery is therefore not needed. Gynecologists also see midwifery as their competitor. We are now under pressure from both sides.” (A midwife working in the labor room)

“Obstetricians have a negative attitude toward midwives. This negative attitude is also induced in residents from the very beginning. I have heard this right from a few residents that they have chosen this discipline to learn cesarean and earn a good income. They also know that the frequencies of natural labor and cesarean section will respectively increase and drop and their market diminish if midwives work efficiently and scientifically. That is why they guard against us.” (A midwifery instructor).

#### Demotivation

As the subcategories of this category, "A sense of worthlessness", "Dishonored midwifery students", "Gap between theory and occupational expectations" and "Attitudes of midwifery instructors" demotivated the learners in different ways.

According to the majority of the participants, "Confusion of midwifery students" caused by inappropriate treatments and environmental condition of clinical education would result in their frustration, despair and loss of self-esteem in the long term.

“In the gynecology clinic, everything was in the hands of the resident and the doctor in the teaching hospital, meaning that midwives are redundant. We would just stand there and watch. We were not even allowed to insert a speculum. There, the midwife played the role of the doctor's secretary. Watching this scene undermined our self-confidence.” (A student).

“Dishonored midwifery students” was another subcategory that caused demotivation. The majority of the students highlighted the derogatory attitudes of obstetricians and residents and even some midwifery instructors toward them in the clinical education setting. A student said,

“Residents treat us and our instructors so badly that we now avoid even talking with them. We have nothing to do with each other. In this way, we avoid friction. We work for them while our spirits are being sunk there.” (A student).

“Humiliation is so much in our discipline that makes us regret choosing it.” (A student).

“A student should hide from doctors. Why should it be like that? I have come as a student here to learn. When the pediatrician comes to neonatal, internal and even maternity wards, we follow him to learn from him, and there is no problem. Yet, when the gynecologist comes to the labor room, they tell us to hide quickly. Or go to the room, the supervisor arrived. Well, she should see me; I am working there. Why should I hide from the gynecologist?” (A student).

In response to these behaviors and existing pressure, midwifery instructors might use displacement as a defense mechanism or drain emotions from the enraging factor to safer subjects such as their students. Some of the students said they had been severely scolded by their instructors for the slightest mistakes they had made:

“One of our clinical problems is that our mistakes are magnified in the presence of patients and other students, which badly lowers our self-esteem. We are therefore constantly afraid of making the same mistake the next time we do the task. We can no longer do it due to so much stress, and we are afraid of making the mistake again.” (A student).

“Warning is very good, as it makes one be careful and learn. We expect them to tell us to be careful and learn to be responsible rather than tell us we cannot do anything and we are not fit to perform midwifery or ask why we have come to the labor room. Well, I (student) have come here to learn something from you.” (A student).

“Gap between theory and occupational expectations” was extracted as another subcategory from the interview texts. This subcategory suggested confusion in midwifery graduates between their theoretical knowledge and occupational expectations despite enriched theoretical teachings in their classrooms. They therefore assumed no future professional applications for their learnt material. An instructor said,

“*What I had learnt was rarely applicable in practice. I think lots of things we were taught can never be used in midwifery, because of lack of context for their application.*” (A midwifery instructor).

“Attitude of midwifery instructors toward midwifery education” was another subcategory and a factor associated with demotivation in teaching–learning in this discipline. According to the interviews with the participants, lack of 24-h midwifery instructors in the hospital, employing uninterested instructors in midwifery, low salaries and poor motivation undermined the commitment and responsibility of some instructors.

“Some midwifery instructors and staff who are uninterested in midwifery or their working conditions have made them uninterested disappoint us; for example, they say that we should have studied nursing, because of its better job market, and instead of showing us how to be employed in the midwifery profession, they constantly dishearten us.” (A student)

Another student said: “*Adjunct faculty members don’t whole-heartedly commit themselves to teaching us; for instance, they say they haven’t yet received their previous year’s salary from the university and we shouldn’t expect them to teach now*.” (A student).

#### Professional nature of midwifery

This theme extracted from the challenges revealed by the participants was mainly associated with the nature of the midwifery profession. The main three categories of this theme included “Community in transition”, “Functional paradox” and “High-risk labor”.

#### Community in transition

Changes associated with a community in transition affect the midwifery profession and even clinical midwifery education. This category comprised two subcategories, i.e. “Changes in attitudes to childbearing” and “Changes in expectations”.

Attitudes to childbearing have recently changed in the community. The changes extracted from the interview texts included reduced childbearing and tendency to actively select the type and place of childbirth. According to the participants, increased frequency of cesarean section and tendency of women to elective cesarean section in private hospitals had reduced learning opportunities for midwifery students. A midwifery instructor said,

“I do not think students will be independently prepared to provide maternal services with this level of training. On the one hand, the frequency of childbirth has dropped. On the other hand, mothers prefer cesarean section as their preferred labor method. Sometimes, students graduate with only a few cases of labor and cannot acquire the necessary experience.” (A midwifery instructor).

“Changes in the expectations of the target community” was another subcategory. Changes in both the position of midwives in the community and the needs of the developing community have affected clinical midwifery education by changing the expectations of the recipients and providers of midwifery services:

“Today, women’s expectations have grown and they rarely consent to give birth in teaching hospitals unless in emergency cases such as preterm labor or referral or other cases of urgency.” (A midwifery instructor)

“Even low-risk mothers visit obstetricians for their prenatal care. Pregnant women in normal conditions don’t need a surgeon. We should screen mothers for their need for specialized services. What percentage of pregnancies and childbirth do actually need the intervention of obstetricians?” (A labor room midwife).

### Functional paradox

Physiological views on labor and childbirth, avoiding interventional practices and providing services based on the individual needs of mothers have been recently recommended while emphasizing evidence-based care approaches. These care services are not, however, completely provided in practice for different reasons. The resulting functional paradox faces students with a conflict between what they have learnt as a physiological labor process and what is actually implemented in hospitals. According to the interviewees, the concepts extracted were classified as three main categories, i.e. “Medical-centered approaches to midwifery”, “Chasm between midwifery theory and midwifery practice” and "Lack of adherence to the clinical guidelines of the Ministry of Health ".

As the main examples of the first category, hasty medical interventions in low-risk labor were extracted from the participant statements.

“Sometimes, a mother is admitted so early that she is already very tired when she reaches 8 cm (end of labor) and labor fails to progress and she is referred for cesarean section. Or, after she had been left in bed for so long, they initiate induction straight away. In fact, they routinely administer medications and syntocinon rather than aiming at physiological labor.” (A student).

“They (residents) expect the ward should be empty. Why on earth should it be empty? Why should a patient be forced to give birth? There is no reason.” (A midwifery instructor).

The majority of the midwifery students and instructors identified a contradiction between what is taught on physiological childbirth and what is clinically observed. Therefore, in the clinical education environment, there is a chasm between midwifery theory and midwifery practice.

"We have never seen a physiological delivery. Why not take a midwifery student for an internship where a physiological delivery takes place? We are told the theory, but we have never experienced it." (A student).

"We have been told that childbirth is a physiological process. We just have to be careful that nothing happens at one time. But we did not see it. Routine induction and stimulation are performed with syntocinon". (A student).

“Lack of adherence to clinical guidelines of the Ministry of Health” obtained as a subcategory in this category showed constantly-performed incorrect procedures and care not based on the mother’s needs in maternity wards to have often turned into routine and experimental habits of the staff and specialized residents despite reliable national and international clinical guidelines on the care of mothers in labor. A midwifery instructor said, “*Sometimes, residents perform inappropriate procedures. I tell my students not to learn whatever they see. These procedures are not evidence-based.*” (A midwifery instructor).

“Here (labor ward) staff say they act according to the national protocol, but when I review the protocol, I see that it’s not the case at all. Our internship should not be performed in hospitals where interventions are routinely used in childbirth.” (A student).

“A few days ago, we saw and talked a lot to a very anxious mother under labor. Our instructor also did so and placed her in different positions. She really felt better. Afterwards, we got ready for her childbirth. The midwife there was impatient, started yelling and shouting, put a lot of pressure on her abdomen and the baby was born, but the mother had ruptures.” (A student).

### High-risk labor

Despite being a physiological process, childbirth can naturally pose many potential risks to those working in this profession and those who are directly involved. Certain labor risks associated with underlying risk factors in the mother significantly increase the risk of pregnancy and childbirth. Midwives should be prepared to deal with certain unpredictable childbirth incidents and complications. According to the participants, this category included two subcategories, namely “High-risk situations” and “Diminished sense of professional belonging”. A midwifery instructor said,

“On the one hand, our job is highly risky. On the other hand, we are not supported either by the hospital or the university. We receive no support, which is a cause for concern. Although the hospital and specialists somehow support the staff if something happens to them, there is no support for me as an instructor. I ask myself what I would have done if this event that happened to the ward midwife had involved me.” (A midwifery instructor).

The incidents and factors cited put some of the educational staff and students off this discipline and profession, and thus diminished their sense of professional belonging. Reasons such as the fear associated with occupational incidents induced in the students by the instructors and ward staff and severe psychological stress related to patient care caused regret about uninformed selection of midwifery and disgust with the profession in the participants. A student said,

“We want to do an intramuscular injection. The instructor scares you for such a simple task and asks why I did it this way. She caused so much stress that I am now stressed even during a simple IV line placement. I know it, but as soon as the instructor comes to watch, I blow it, or the vein ruptures, or when I try to establish an IV line, blood splashes out.” (A student).

“I liked midwifery very much since school days and always said I would study midwifery. I still like it but expected my interest to grow many times rather than to diminish when I came to the university.” (A student).

A midwifery instructor said,

“When a student sees that a resident talks to and treats my instructor so badly, she asks herself what her future will be like. Anyway, I am her (student’s) future. It ruins her motivation. This significantly affects students’ disinterest in their profession.” (A midwifery instructor).

According to the participants, in the absence of necessary support for the reasons discussed, midwifery instructors and students will face a double risk in dealing with the risks of childbirth, which can decrease their interest in the midwifery profession.

## Discussion

According to the present findings, discriminatory approaches to the midwifery profession in the health system manifesting itself as “educational inequalities” and insecure position of educational professionals in hospitals have faced clinical education with challenges and demotivated the instructors and students in two midwifery schools in Iran. In the face of potentially high-risk labor, failure to receive legal support in cases of occupational incidents makes both the students and instructors feel unsecure and their challenges are complicated by the attitude of the community in transition toward childbearing and functional contradictions of the maternity staff between medicalization and physiological views on labor.

“Discriminatory approach to the midwifery profession” was extracted as the first theme. Despite the vital role of midwives in saving the economic resources of the health system, providing families with quality services and improving maternal and neonatal health indicators, the discriminatory approach of the health system to different dimensions of the midwifery profession has caused the potential of this profession to be neglected, has deprived midwives of the opportunity to independently provide services and has yielded their insecure position. Moreover, despite the clinical competence of midwives as per international standards, lack of autonomy makes them perceive loss of control over their job. Professional control refers to a combination of skilled discretion or the creativity required for performing tasks and decision autonomy or organizational opportunities for decision-making on work [18]. Professional control is known as the main predictor of the inclination to carry on working and vice versa; in its absence, nurses tend to leave their job [19]. In addition, midwifery students’ identification of the lack of functional independence in the midwives working in hospitals make them feel vulnerable and insecure [20]. Research suggests unequivocally defining professional autonomy, receiving support from managers and teamwork in midwifery promote professional autonomy [15, 16].

Educational justice refers to equally sharing educational facilities among all students of universities of medical sciences regardless of their academic discipline. To achieve this goal, measures should be adopted to provide appropriate conditions and facilities in a fair manner, employ capable faculty members and fill the gap between practical courses and clinical practice. This approach was, however, found to be largely neglected in midwifery education as per the interviews, especially the FGDs [21]. Ignoring the clinical competence of midwifery instructors in this educational atmosphere has also reduced their autonomy over time. Given that instructors’ autonomy is essential for promoting confidence, reverence and clinical skill and competence, its absence undermines the eagerness required for teaching [22] and provokes anger, apathy and sense of worthlessness in midwifery students.

Safe and indiscriminate educational environment has been long focused and discussed by instructors, professionals and educational organizations. Research is also ongoing on improving medical education [23]. An inappropriate and suboptimal educational environment is associated with inadequate patient care and improper learning outcomes [24–26].

The participants’ concerns included unbalanced distribution of power and conflicts of interest. The theory of birth territory and midwifery guardianship is partly associated with the way power is applied in organizations. According to this theory, the structure and function of contemporary midwifery services make midwives obedient or subordinate to doctors, which undermines the eagerness required for teaching [27]. Socioeconomic and legal power networks have gained control of birth territory. Recent financial incentives provided to promote natural childbirth in Iran have caused numerous conflicts of interest between gynecologists and midwives performing labor in private hospitals. Furthermore, the growing tendency to elective cesarean section prior to the banning of non-emergency cesarean section dramatically increased the rate of primiparous cesarean section over the last two decades. Despite imposing policies that promote natural childbirth in Iran, the rate of cesarean section in this country still ranges from a minimum of 47.9% to 87% in some private hospitals [28, 29].

Rather than professional skills of staff, the organizational culture governing birth territory appears to constitute the main predictor of positive professional interactions [30]. An inappropriate organizational environment is associated with high stress levels, burnout, depersonalization and emotional exhaustion in both students and instructors of medical sciences [24, 25]. It also creates cognitive overload and thus limits both learning opportunities and the time required for rethinking and discussion and adversely affects the quality of clinical education [26]. Educational environments should be made appropriate in terms of their physical aspects, facilities, educators and staff. A cooperative atmosphere also helps learners fulfill their goals [31]. Moreover, a non-supportive educational environment was referred to as the phenomenon of vertical violence against students [32]. Creating a safe environment based on friendly behaviors, mutual respect and clinical reverence for students can induce a sense of self-worth in midwifery students and improve teaching and learning conditions [13]. Moreover, supportive behaviors of staff and cooperation of physicians with students facilitate their clinical education and professional development and affect the learning/teaching process [33].

The unwillingness of instructors, especially the experienced ones, to provide services in inappropriate and unsupportive environments deprives students of independent functioning at the client bedside and decreases the self-confidence required for their functional independence [34]. In contrast, employing experienced instructors to provide students at the beginning of clinical education with clinical guidelines, including internship behavioral goals, ward regulations, job description of students and required references, causes satisfaction in students, prevents their confusion, helps them comprehend their occupational responsibilities and duties, more efficiently lean clinical skills and apply theoretical learning to clinical practice [35]. Creating a facilitating environment based on intra-organizational coordination among faculties and hospitals can therefore help promote clinical midwifery education.

The consequences associated with the nature of the midwifery profession was obtained as the second theme. A community in transition during its period of shift from one status to another is characterized by changes in attitudes toward childbearing and changes in the ideal care expected from care providers by women such as painless and uncomplicated childbirth. These changes also include decreased fertility rates, having the choice of the method and place of labor and significant reductions in the frequency of natural birth and referrals, especially to crowded public and teaching hospitals.

Decisions on pregnancy and maternity care are made or changed over time through a dynamic and temporal process depending on socioeconomic and political developments in a country and based on political approaches to population control. At the forefront of communication with women of reproductive age, motivated and skilled midwives play an undeniable role in aligning the childbearing decisions of women with national population policies [36].

The revised midwifery educational curriculum accords with the standards recommended by the ICM and midwifery care model, in which, attitude to pregnancy and childbirth is regarded as a natural stage in life and women-centered cares are provided based on their needs. This type of care is based on avoiding routine care and unnecessary interventions in low-risk labor. In contrast, labor is generally managed in clinical educational settings under the direct supervision of an obstetrician and using a medical approach. Hasty labor and medical interventions abundantly observed in these educational environments cause a functional paradox in midwives and students, suggesting the redundancy of midwives given that childbirth is mainly managed by physicians [37]. Comparing midwives with obstetricians in terms of the care they provide in low-risk labor showed that low-risk childbirth performed by midwives is associated with decreases in cesarean Sect. (30%-40%), interventions and operative labor [38]. Despite being naturally regarded as a physiological process, childbirth can entail risks, unforeseen events and inevitable incidents. Despite the key role of sense of belonging to the midwifery profession in learning, inducing fear in students by instructors and other personnel and high stress levels in this highly-risky environment undermine this sense in the students [39]. Many midwifery students select their discipline before adequately understanding their profession and the history of the work performed in this discipline [40]. High levels of psychological stress was reported in 56% in one study on Iranian midwifery students. Their main dimensions of stress included unpleasant emotions, humiliating experiences, the suffering caused by observing patients in critical conditions and the factors associated with instructors [12]. High levels of stress in these students might have been caused by different stressors such as the gap between theoretical knowledge and clinical practice. Although psychological stress and its associated factors can differently affect students based on their discipline, nationality and region [41], these factors can reduce their interest in midwifery and even lead to their withdraw from the job. In addition, retaining students in the educational program and training qualified and committed health workers are regarded as a key component of health programs [40].

## Conclusion

The experiences of the participating midwifery instructors and students suggested numerous problems in the clinical environment of midwifery education. The findings obtained in the present study cannot be transferable. It is therefore recommended that further similar studies be conducted on midwifery education in other universities in Iran. Other studies in Iran are consistent with the present study and suggest midwifery education is not aligned with the education provided for many medical professions. This fact not only increases negative attitudes and a sense of discrimination in midwifery students, but also demotivates them and significantly limits their learning opportunities. Laying plans to modify the unfair structure of the systems, promote justice in existing clinical educational environments, expedite inter-professional cooperation and create learning opportunities for midwifery students in dedicated environments is therefore crucial for teaching safe midwifery care. It is recommended that capable instructors who are interested in the profession be employed and their active presence and participation in decision-makings be facilitated in clinical educational environments to improve the learning experience of midwifery students.

## Limitation and strength

Although the present results can partly reflect problems in midwifery education in the study setting, an unbiased in-depth investigation of the participants' experiences in a safe environment constituted a strength of this study.

Also, the rigor and trustworthiness of the study data were observed based on “the Lincoln and Guba criteria. To confirm credibility, the transcripts of the interviews were reviewed by the participants (member-check), prolonged engagement of the researcher with the data was performed, the codes and categories were independently controlled by the research team. To confirm dependability using the code-recode procedure, several interviews were re-coded by the researcher two weeks after the initial coding and rarely-observed contradictions were resolved through a review performed by the research team. To confirm transferability, maximum diversity was observed in selecting the participants in terms of their personal and occupational details and efforts were made to inform the readers of the study methods, time and location by comprehensively detailing the stages, i.e. sampling, interviews and FGDs. An external audit was performed by two outside observers, including a midwifery faculty member and a PhD graduate in reproductive health, as experts in midwifery education and qualitative research using the documented objectivity of the findings, transcripts of the interviews and the analyzed data. To ensure confirmability, the results of three interviews were made available to two researchers in midwifery acquainted with qualitative research analysis, and they were asked to independently code these interviews and investigate coding contradictions. To confirm authenticity and determine potential bias in expressing the data and conveying conflicting views as they were, eligible participants were selected from diverse age groups, education levels and work experiences, and their quotes were completely described to enhance the readers’ understanding. Furthermore, the researchers made efforts to prevent their preconceived beliefs from affecting the collection and analysis of the data.

## Data Availability

The datasets generated and analyzed during the current study are not publicly available due to [Confidentiality of participants' faculty names] but are available from the corresponding author on reasonable request.
